# Bladder stone formation due to neglected double J stent: a case report

**DOI:** 10.1097/MS9.0000000000001294

**Published:** 2023-09-27

**Authors:** Ali Jawad, Hussein Hamdar, Ali Alakbar Nahle, Hussein Taher, Ali Faisal Ahmad, Adnan Ahmed

**Affiliations:** a Faculty of Medicine, Damascus University; b Faculty of Medicine, Al-Sham Private University; c Department of Urology, Al Assad University Hospital, Damascus University, Damascus, Syria

**Keywords:** case report, double J stents, encrustation, large bladder stone

## Abstract

**Introduction and importance:**

Double J (DJ) stents are commonly used in genitourinary procedures, but they can lead to complications including infection, hematuria, encrustation, and stone formation. The longer the duration of encrustation, the greater the risk of complications and renal dysfunction. Forgotten stents pose challenges for patients and can require endourological or open surgical procedures.

**Case presentation:**

A 40-year-old man with hypertension and coronary artery disease had a forgotten DJ stent for 3 years, causing suprapubic pain and dysuria. Kidney, ureter, and bladder (KUB) revealed a coiled DJ stent with a large bladder stone and encrustation, and an open cystolithotomy was successfully performed. Recovery was uneventful, and the patient was discharged without complication.

**Clinical discussion:**

Ureteral stents, including the DJ stent, are commonly used for urological conditions but can cause complications if retained beyond the intended timeframe. Optimal timing for stent removal is crucial, and patients’ healthcare knowledge and adherence are critical to preventing retention. KUB X-ray can evaluate stent encrustation and bladder stones. Cystoscopy is the typical approach for stent removal, but supplementary interventions may be necessary. Open surgery was recommended for removing a large bladder stone and encrusted stent in this case.

**Conclusion:**

Timely removal of DJ stents is crucial to avoid complications. Extended retention can cause problems such as encrustation and stone formation. Patient education and adherence are essential to prevent retention and forgetfulness. This case report highlights the importance of careful management of patients with DJ stents for optimal outcomes and prevention of complications.

## Introduction

Highlights

- Double J (DJ) stents can cause complications if retained beyond the intended timeframe, and timely removal is crucial to avoid complications.
- Patient education and healthcare adherence are essential in preventing retention and forgetfulness of DJ stents.
- Imaging techniques such as Kidney, ureter, and bladder X-ray can evaluate stent encrustation and bladder stones, and cystoscopy is a typical approach for stent removal.
- This case report emphasizes the importance of careful management of patients with DJ stents for optimal outcomes and prevention of complications.

Double J (DJ) stents have been widely used in genitourinary procedures^1^. Recent advancements in stent design and materials have contributed to their improved utilization. However, complications associated with these stents, including infection, hematuria, encrustation, and stone formation, have become more severe compared to previous cases. The formation of large bladder stones and encrustations can significantly complicate the patient’s condition and result in renal dysfunction^2^. While large bladder stones are relatively uncommon, the severity of complications increases with the duration of encrustation^2^. The safe duration for keeping a stent without complications has not been fully determined. However, surpassing the initially planned duration or neglecting to remove it is considered unsafe and life-threatening^3,4^. Managing forgotten stents presents challenges for patients, and both endourological and open surgical procedures can encounter difficulties and complications associated with the stent^4^. In this case report, we present an exceptional case of a massive bladder stone that developed as a result of a neglected DJ stent for a period of 3 years. The stone was successfully managed through open surgical intervention.

This work has been reported in line with the SCARE 2020 criteria^5^.

## Case presentation

A 40-year-old Syrian male patient, who is a smoker and has a medical history of hypertension and coronary artery disease, was admitted to the hospital with complaints of suprapubic pain and dysuria. The patient did not present with fever, nausea, nocturia, urinary incontinence, vomiting, constipation, or decreased appetite. Three years ago, the patient underwent DJ stent insertion for stone removal, but due to a lack of medical awareness, follow-up was not maintained. There is no reported history of genital discharge or sexually transmitted disease.

During the physical examination, the patient presented with suprapubic tenderness. Vital signs indicated a blood pressure of 125/90 mmHg, a pulse rate of 100 beats per minute, a respiratory rate of 18 breaths per minute, and a temperature of 37.1°C. Blood tests yielded normal results, and the serum creatinine level was measured at 1.2 mg/dl. The urine test came back normal, except for the microscopic examination, which revealed the presence of calcium oxalate. The urine culture detected *Escherichia coli*, which was successfully treated with antibiotics.

**Figure 1:**
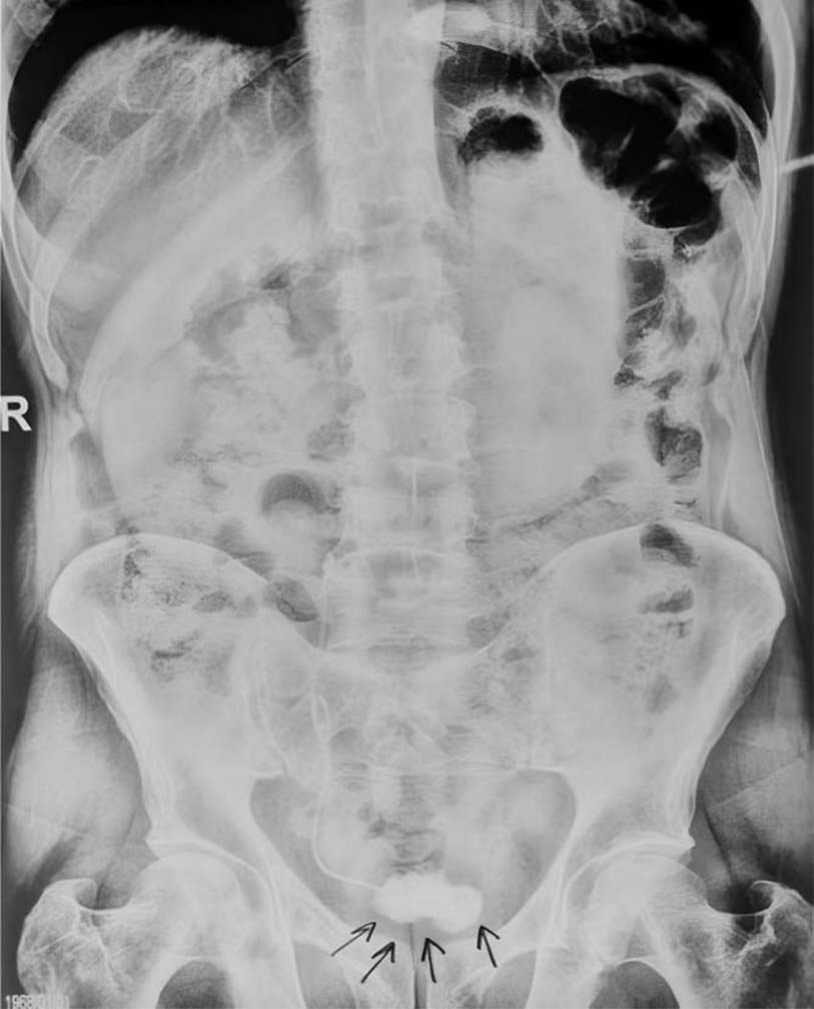
Preoperative kidney, ureter, and bladder showing entire coiled double J stent with a stone in the bladder in contact with it accompanied by encrustation.

A photograph of the kidney, ureter, and bladder (KUB) demonstrated a coiled DJ stent located in the right lower lumbar and sacral paravertebral space, accompanied by a 40 × 30 mm bladder stone in direct contact with it, along with encrustation (Fig.1). Unfortunately, due to limited hospital resources, financial constraints, and the consequences of war in our country, computed tomography (CT) scans and pelvic ultrasound were not feasible.

**Figure 2:**
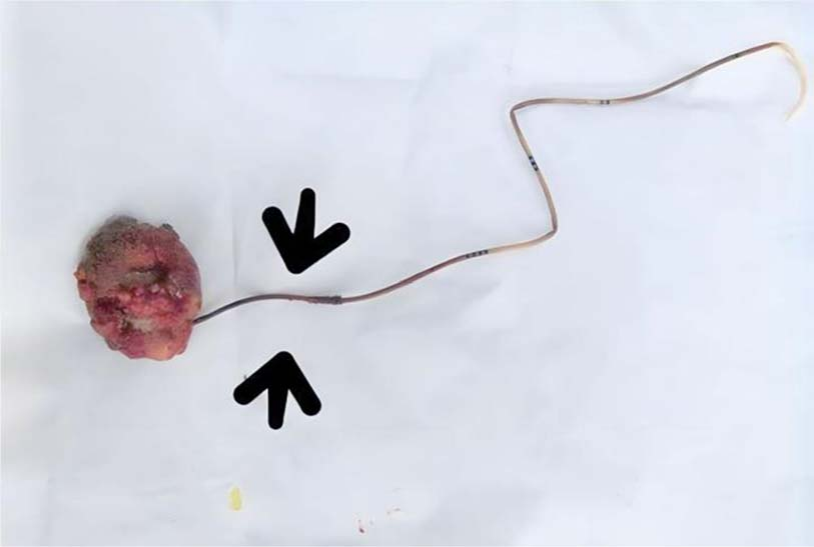
Removed double J stent and bladder stone accompanied with encrustation.

The patient was diagnosed with a neglected DJ stent accompanied by a bladder stone and subsequently underwent an open cystolithotomy procedure under spinal anesthesia while positioned supine. During the procedure, a Foley catheter was inserted into the bladder, and normal saline was introduced. An incision was made on the ventral midline pelvis between the rectus muscles, followed by a vertical incision of the detrusor muscle. The bladder was then opened, allowing for the removal of both the DJ stent and the stone (Fig. 2). The bladder closure was achieved using absorbable sutures, and a three-way catheter was inserted through the urethra into the bladder for urine drainage and bladder irrigation as needed.

The patient’s postoperative period was uneventful, with close monitoring by the healthcare team to ensure proper healing and minimize potential complications. Effective pain management was provided with diclofenac 50 mg orally twice a day for one week, along with antibiotic coverage using ciprofloxacin 500 mg orally twice a day for one week and rocephin 1 g intravenously twice a day for 3 days. The urethral catheter was removed on the second postoperative day, and the patient exhibited normal urinary output. With good overall health and the absence of pain or discomfort, the patient was discharged on the second postoperative day without any complications.

## Discussion

Over a century ago, Shoemaker documented the initial application of a ureteral catheter, marking the beginning of the use of ureteral stents for a wide range of urological conditions. Initially, these stents were employed in the treatment of urinary tract issues, such as ureteral or renal stones, renal transplant surgery, and reconstructive surgery of the upper urinary tract, utilizing various surgical methods including endourological, laparoscopic, robotic, or conventional approaches. Therapeutically, these stents served multiple purposes, including relieving obstructive uropathy and providing conservative treatment for upper urinary tract trauma^6,7^. In our specific case, the DJ stent was implanted as a therapeutic intervention for managing renal calculi. The term ‘Double J’ refers to the prevailing stent design that was first introduced by Finney in 1978. Since then, different biomedical companies have developed stents with varied structures primarily to mitigate issues such as encrustation and infection, improve urine drainage, and minimize the impact on patients’ quality of life^8^.

The exact duration for which a stent can be safely retained remains uncertain, and exceeding the intended timeframe or neglecting its removal is considered highly risky and potentially life-threatening^3,4^. However, another study recommends replacing or removing a DJ stent within a period of 6 weeks to 6 months to prevent complications^9^. Therefore, further research is needed to establish the optimal timing for DJ stent removal without complications. Prolonged stent retention can lead to severe complications such as migration, fragmentation, encrustation, and stone formation^9^. Encrustation refers to the formation of mineral crystals on the surfaces of a ureteral stent, both internally and externally. This process can result in significant complications, especially when stents are left in place for extended periods or forgotten^10^. The main microorganisms responsible for bacterial biofilm development and subsequent encrustation are *E. coli*, *Streptococcus*, and *Pseudomonas* ^11^. In our patient’s case, the urine culture indicated the presence of *E. coli*, significantly increasing the risk of biofilm formation and subsequent encrustation. Additionally, bladder stones, which typically measure less than 1 cm and often pass out of the body naturally, can be a complication^12^. However, in our specific case, a large bladder stone measuring 4 cm was detected, highlighting the potential development of sizable bladder stones as a complication resulting from prolonged DJ stent placement.

Inadequate understanding of healthcare matters, which can lead to reduced patient adherence and comprehension, has been identified as a contributing factor to an increased risk of stent retention and forgetfulness^13^. In the present case, the patient neglected the stent for a period of 3 years after undergoing stone surgery due to lack of awareness, financial constraints, and transportation difficulties, resulting in the development of a bladder stone and encrustation on the exterior of the stent. This emphasizes the crucial importance of enhancing patients’ healthcare knowledge and adherence by regularly consulting healthcare professionals and ensuring the timely removal of the stent.

The presentation of a forgotten stent can manifest in various ways. Damiano *et al*.^14^ reported that 25.3% of cases presented with flank pain, while 18.8% exhibited irritative bladder symptoms, similar to our patient. Additionally, hematuria was observed in 18.1% of patients and fever in 12.3%. It is widely acknowledged that asymptomatic patients are more prone to neglect or disregard their stents, resulting in a higher prevalence of forgotten stents within this particular subgroup^15^.

While imaging is generally not necessary for the removal of most stents, patients at a higher risk of encrustation may benefit from imaging to assess the severity and location of encrustation on the stent. Standard KUB X-ray is often sufficient to provide information about the stent and the extent of encrustation. However, in more complex cases, an ultrasound or CT scan may be necessary to accurately evaluate the degree of encrustation and establish a comprehensive stent removal strategy^16^. In terms of evaluating bladder stones, radiograph or KUB film is commonly used as the initial diagnostic test. Nevertheless, computed tomography (CT) or bladder ultrasonography are reliable alternatives for imaging potential bladder stones^17^. In our specific situation, we relied solely on KUB X-ray as it was adequate for evaluating our patient’s condition, given the unavailability of a CT scan due to technical issues and limited funding in our hospital.

The typical approach for removing a DJ stent involves a cystoscopy procedure, during which a small camera is inserted into the bladder through the urethra to locate and extract the stent^18^. However, if the stent has become encrusted or embedded in the bladder wall, its removal can present greater challenges, requiring additional interventions such as laser lithotripsy or open surgery. The treatment method for a bladder stone depends on its size and location. Smaller stones can be dissolved using medication or managed with non-invasive techniques like ESWL (extracorporeal shock wave lithotripsy), while larger stones may require more invasive procedures such as cystolitholapaxy or open surgery^9,19^. Considering the significant size of the stone, the presence of encrustations, and the lack of suitable materials, open surgery was recommended as the appropriate course of action for this patient in our specific case. Furthermore, in our case, non-steroidal anti-inflammatory drugs (NSAIDs) were prescribed to ensure the safe and efficacious management of postoperative pain. It is worth noting that a previous study emphasized the importance of administering NSAIDs to all postoperative patients unless contraindicated^20^. Prophylactic antibiotics were used in our case, supported by literature indicating a higher incidence of urinary tract infections and bacteremia following urological procedures. This approach effectively prevents postoperative complications in transurethral urological surgeries^21^.

## Conclusion

In conclusion, the presented case underscores the significance of the timely removal of DJ stents and the potential complications that can arise from prolonged retention or neglect. Ureteral stents, such as the DJ stent, have played a crucial role in managing urological conditions, including renal calculi. However, prolonged stent retention can give rise to complications such as encrustation, stone formation, and migration. The optimal timing for stent removal remains uncertain, necessitating further research to establish guidelines for preventing complications. Patient education and adherence are vital in preventing stent retention and forgetfulness, highlighting the importance of regular consultations with healthcare professionals.

## Ethics approval

Ethics approval was not required for this case report at our institution, the Faculty of Medicine at Damascus University, Damascus, Syria.

## Consent

Written informed consent was obtained from the patient for publication and any accompanying images. A copy of the written consent is available for review by the Editor-in-Chief of this journal on request.

## Sources of funding

Not applicable.

## Author contribution

A.J. is the first author: contributed to drafting, reviewing, and editing; H.H. is a co-first author: contributed to drafting, reviewing, and editing, and bibliography; A.A.N.: contributed to drafting, reviewing, editing, and corresponding; H.T.: contributed to drafting, reviewing, and editing; A.F.A.: contributed to reviewing, editing, and supervising; A.A.: contributed to reviewing, editing, and supervising. All authors read and approved the final manuscript.

## Conflicts of interest disclosure

The authors declare that they have no conflicts of interest.

## Research registration unique identifying number (UIN)

This study is a case report, so we cannot register it as a trial.

## Guarantor

Dr Adnan Ahmed.

## Data availability statement

Not applicable.

## Provenance and peer review

Not commissioned, externally peer-reviewed.
